# Now or Later? Stress-Induced Increase and Decrease in Choice Impulsivity Are Both Associated with Elevated Affective and Endocrine Responses

**DOI:** 10.3390/brainsci11091148

**Published:** 2021-08-29

**Authors:** Lisa Simon, Talita Jiryis, Roee Admon

**Affiliations:** 1 School of Psychological Sciences, University of Haifa, Haifa 3498838, Israel; lisazelen@gmail.com (L.S.); talitakumi17@gmail.com (T.J.); 2 The Integrated Brain and Behavior Research Center (IBBRC), University of Haifa, Haifa 3498838, Israel

**Keywords:** acute stress, delay discounting, impulsivity, affect, cortisol, individual differences, quadratic, inverted U

## Abstract

Exposure to acute stress elicit physiological and psychological responses that can impact decision-making, often expressed as an increased tendency to act in an impulsive manner following stress. Delay discounting (DD) task has emerged as a reliable measure of impulsive behavior in the form of choice impulsivity (CI). Interestingly, studies that examined the effect of acute stress on DD performance reported mixed results. To address this, we conducted a within-subject examination of the impact of acute stress on CI, focusing on individual differences in response patterns. One hundred and fifty healthy female participants completed the DD task twice, before and after undergoing an acute laboratory stress induction procedure. Saliva samples and self-report mood and affect measures were collected at four time points throughout the session. Fifty-nine matched healthy control participants completed only the DD task twice, with no stress in between. Results indicate that the acute stress procedure elicited the expected effects of increased cortisol release and increased negative mood and affect, at the group level. With respect to DD, stress indeed increased CI at the group level, yet participants differed in the magnitude and direction of this effect. Interestingly, regression analysis revealed quadratic relations between stress-induced changes in CI and cortisol release. Indeed, dividing the sample into three sub-groups based on the impact of stress on CI revealed that, compared to participants that exhibited no substantial change in their CI following stress, participants that exhibited either stress-induced increase or decrease in their CI also exhibited more stress-induced cortisol release, as well as more negative affect. Taken together, these findings suggest that elevated physiological and psychological responses to stress are associated with either increased or decreased choice impulsivity, thus depicting quadratic relations between stress and impulsivity.

## 1. Introduction

Encounter with a stressor, a situation that involves real or perceived threat to homeostasis, elicits a complex cascade of physiological responses aiming to maintain/reestablish the threatened homeostasis (i.e., the stress response) [1,2]. These physiological stress responses, in turn, induce short- and long-term effects on individuals’ emotional state and cognitive performance. In the context of decision-making, experimental data mostly suggest that acute stress may lead to suboptimal choice selection. Under stress, individuals often fail to adhere to rational choice models that assume that decisions are based on the weighing of the utilities and probabilities associated with all available courses of action [3]. A prominent example of such impact of stress on decision-making is an increased tendency to act in an impulsive manner following stress, disregarding more long-term goals. For example, acute stress was shown to increase craving for smoking and alcohol use [4,5], and levels of perceived stress predicted smoking relapse in women [6]. Similarly, acute exogenous cortisol administration was found to negatively impact decision-making and was related to increased risky choices [7].

Over the last two decades, delay discounting (DD) task has emerged as a reliable laboratory measure of impulsive behavior in the form of choice impulsivity (CI) [8,9]. In a typical DD task, participants are requested to choose between a smaller immediate vs. a larger delayed monetary reward. The rate at which individuals discount future rewards varies as a function of individual and contextual factors, with larger discounting rates considered a marker for CI (inability or unwillingness to wait for larger future rewards) [10,11,12]. Individuals exhibiting behavioral patterns that are associated with impulsive choice selection such as substance abuse, smoking and pathological gambling, demonstrated higher discounting rates in the DD task compared to healthy controls [13,14,15]. Interestingly, studies that specifically assessed the impact of stress on CI using the DD task reported mixed results. Several studies reported more impulsive choices (i.e., higher discounting rate) under stress [16,17], or in response to acute exogenous cortisol administration [18]. Other studies found higher CI under stress only in a subset of participants that presented elevated cortisol response [19], only in females [20], or only in individuals with low trait perceived stress [21]. Additional studies found no effect of stress on DD task performance [22,23,24].

One potential account for this lack of clarity may stem from the fact that despite substantial inter-individual differences in physiological and psychological stress response patterns [25], previous studies often focused on group mean effects, or at most assessed linear or binary relations in the impact of stress on CI. This approach does not take into consideration vast literature that ties impaired emotional and cognitive functioning in psychopathological populations with amplified but also blunted physiological and endocrinological stress reactivity patterns [26,27,28]. Along a similar line, among healthy females, both hyper and hypo cortisol responses to acute stress were shown to be associated with elevated negative affect compared to moderate cortisol response, implying a quadratic pattern [29]. Gender differences in the impact of acute stress on impulsivity may represent an additional contributing factor for reported inconsistencies in the literature. To this end, gender differences have been observed in both endocrine [30,31] and behavioral responses to acute stress [32,33]. Even specifically in the context of DD task, acute stress led to elevated CI in females but not males [20]. Following these, and additional findings, it has been suggested that gender should be controlled in studies assessing the impact of stress on CI [19]. Another factor that may contribute to the mixed results is variability in study design, as prior studies implemented the DD task after the stress induction [17,21,24], after administration of hydrocortisone [18], or during a threat of shock [23]. One study measured DD rates before and after stress using a questionnaire [19], while another study measured DD rates before the stress and performed the stress manipulation itself on the next day [22]. Considering substantial individual differences in CI regardless of stress, as indicated above, a within-subject design may offer a preferable approach to specifically probe stress-induced changes in CI. To our knowledge, no study to date has investigated the effect of acute stress on CI by measuring performance in the DD task both before and after exposure to stress.

The aim of the current study is to fill this gap and assess the impact of acute stress on CI, while focusing on individual differences in these effects and their associated physiological and emotional patterns. For that, one hundred and fifty healthy female participants were recruited to undergo a behavioral experimental session during which they completed the DD task twice, before and after undergoing an acute laboratory stress induction procedure. Affective and endocrine (i.e., cortisol) responses were assessed at four time points throughout the session, accounting for before, during and after stress exposure. Fifty-nine matched healthy controls completed only the DD task twice, with no stress in between. Based on the literature cited above we hypothesized that participants would exhibit an increased tendency to act in an impulsive manner following stress (i.e., elevated CI), yet that the magnitude and direction of this effect would greatly differ among individuals. We further hypothesized that individual differences in the impact of stress on CI will relate to variability in affective and endocrine responses to stress, yet that these relations will not be linear nor binary but rather quadratic. 

## 2. Materials and Methods

### 2.1. Participants 

Two hundred and nine healthy female participants were recruited to the study (mean age 25 ± 3.4, range 20–36). Only female participants were recruited in order to overcome the above-mentioned gender-related variability. All participants completed online screening questionnaires prior to their experimental session. Inclusion criteria included right-handedness and normal or corrected vision. Exclusion criteria included any acute or chronic illness, current use of any medication, current or past neurological or psychiatric disorders including attention deficit hyperactivity disorder (ADHD) or learning disorders. The study protocol was approved by the ethical committee of the University of Haifa (approval number 029-17). Written consent was obtained from all participants, and participants received monetary compensation for their time. 

### 2.2. General Procedure

**Figure 1:**
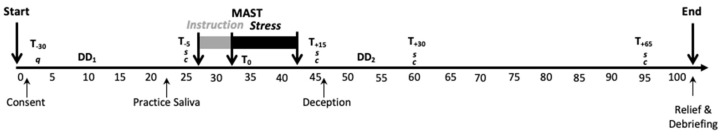
A detailed timeline of the stress experimental session. DD—delay discounting task; MAST—Maastricht acute stress test; q STAI-T—state and trait anxiety inventory, BIS—Barratt impulsiveness scale and PSS—perceived stress scale; s PANAS—positive and negative affect and VAMS—visual analogue mood scale; c Cortisol.

All experimental sessions took place between 12:00 and 16:00 p.m. in order to minimize diurnal variations in cortisol levels [34]. Further, to control for saliva collection, participants were asked to refrain from alcohol or coffee intake and not to eat, smoke, exercise, or brush teeth one hour prior to the experiment. After arriving at the laboratory, participants received information about the study and the measurements that would be taken and were asked to provide written informed consent. One hundred and fifty participants that were assigned to the stress condition then completed self-report questionnaires of trait anxiety (State-Trait Anxiety Inventory, STAI-T; [35]), perceived chronic stress (Perceived Stress Scale, PSS; [36]) and trait impulsivity (Barratt Impulsiveness Scale, BIS; [37]). Next, these participants completed their first session of the DD task (DD_1_), which was followed by baseline (T_−5_) measures of state affect (Positive Affect and Negative Affect Schedule, PANAS), mood (Visual Analog Mood Scale, VAMS), and saliva. After this, participants were told that another experimenter would continue the session. In fact, the experimenters were changed in order to achieve a more reliable stress-induction procedure. The new experimenter operated the Maastricht Acute Stress Test (MAST) that takes approximately 15 min to complete (see detailed explanation below). Immediately following MAST completion, affect, mood and saliva were assessed for the second time (T_+15_). Next, participants completed the second session of the DD task (DD_2_), and then the third affect, mood and saliva assessment (T_+30_). Thirty-five minutes later participants completed their fourth and final, affect, mood and saliva assessment (T_+65_), and then they were debriefed about the stress manipulation and compensated for their time. See Figure 1 for a detailed timeline of the stress experimental session. A control group of fifty-nine matched healthy control participants completed only the part of the DD task twice from this experimental session, without any exposure to stress in between. The control group was recruited in order to assess the stability of behavioral responses in the DD task when performed twice with no stress induction in between. Accordingly, no affective and endocrine measures were collected from the control group. The assignment to experimental vs. control group was random and the two groups were matched with respect to the fact that participants in both groups were all healthy young females that passed identical exclusion/inclusion criteria.

### 2.3. Measures

#### 2.3.1. Delay Discounting (DD)

**Figure 2:**
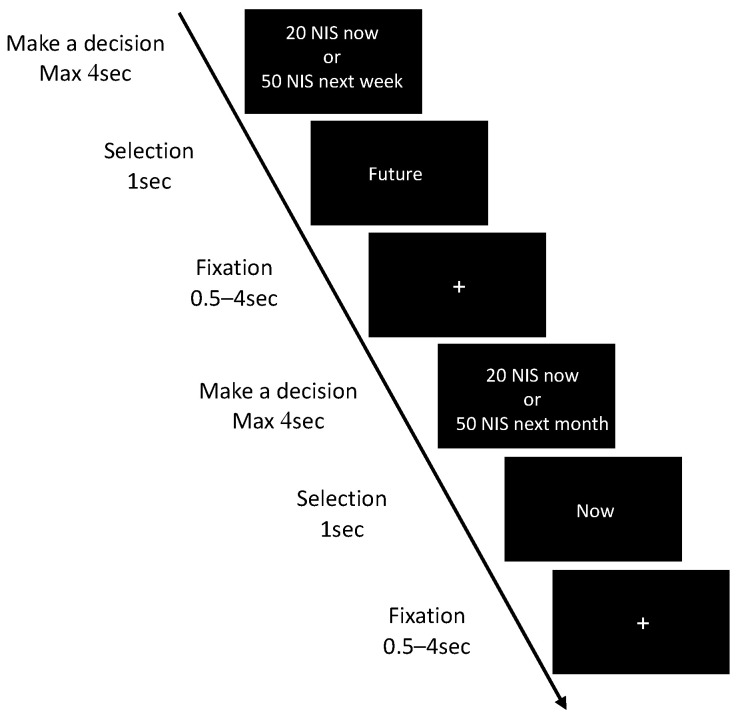
Layout of the delay discounting (DD) task. This well-established task assesses behavioral indices of choice impulsivity (CI) by asking participants to decide whether they want 20 NIS right now (at the end of the experiment) or one of 10 larger monetary amounts after one of six delays. Each combination of an amount and delay appeared once in a randomly counterbalanced order across the sixty task trials. NIS—New Israeli Shekels.

The DD task is a well-established behavioral measure for CI [38]. The current version of the task was developed in our lab and included six training trials prior to one block of sixty test trials. Each trial involved a choice between two monetary rewards that differed with respect to the amount of money and the delay until delivery. In all trials, an immediate smaller monetary reward of 20 NIS that is available now (at the end of the study) was presented on the upper part of the computer screen, while a delayed future reward was presented on the lower part of the screen. Future rewards varied in their amount (one of ten amounts: 20, 25, 30, 35, 42, 50, 75, 100, 150 and 200 NIS) and delay until delivery (one of six delays: one week, two weeks, one month, three months, six months and one year). Each combination of amount and delay appeared once in a randomly counterbalanced order across the sixty trials. The instruction cues for “now” vs. “future” choices were the 1 or 3 keyboard keys, counterbalanced across participants. Participants had up to four seconds to make a decision, following which their selection appeared on the screen for one second (“now”; “future”; “no response”). Trials were separated by 0.5–4 s of fixation. See Figure 2 for a layout of the DD task. Participants were instructed to carefully weigh up each decision and were told that one randomly selected monetary choice will be paid to them at the end of the experimental session in order to increase the validity of their choices throughout the task.

#### 2.3.2. Maastricht Acute Stress Test (MAST)

The Maastricht Acute Stress Test (MAST) is a well-established laboratory acute stress procedure that was shown before to yield robust endocrine and affective stress responses among healthy adults [39]. The task starts with a five-minute preparation phase during which participants are instructed about the task. In order to create an additional aspect of stress, during that time participants are also asked to fill a consent form allowing us to videotape their pain facial expressions. In practice, facial expressions were not recorded. The subsequent ten-minute acute stress phase includes several exposures to cold pressure stress that are interleaved with a mental arithmetic challenge in the form of counting backward as fast and as accurately as possible in steps of 17 starting from 2043. The mental arithmetic challenge is also accompanied by social-evaluative pressure of negative feedback on participants’ performance, feedback that is provided by the experimenter regardless of participants’ actual performance. The MAST protocol also involves uncontrollability and unpredictability as participants are informed that the computer randomly chooses the order and duration of the cold pressure and mental arithmetic trials. In fact, the duration and order of cold pressure stress stimuli and arithmetic trials are fixed for all participants. Following MAST completion participants were told that due to their poor performance they will need to repeat the task at a later stage (i.e., deception), thus denying their relief in order to prolong the effect of the acute stressor [29]. Upon session completion, participants were informed that repeating the task was not necessary since their performance was good enough (i.e., relief and debriefing). 

#### 2.3.3. Cortisol Saliva Samples 

Saliva samples were obtained at four time points throughout the session (T_−5_, T_+15_, T_+30_, T_+65_ with respect to stress onset) by placing a cotton swab in participants’ mouths for 45–60 s using Salivette collection devices (Sarstedt, Nümbrecht, Germany). Samples were stored at −20 °C until analysis. After thawing, saliva samples were centrifuged for 10 min at 4000 rounds per minute (rpm) to remove particulate material. Cortisol concentrations from saliva samples were assayed using a solid-phase enzyme-linked luminescence immunoassay at the Cognitive Psychology Department, University of Hamburg, Germany (Director: Prof. Lars Schwabe). The assay was conducted with 50 µL of saliva according to the specification and protocols of the manufacturer (LIA; IBL/Tecan, Hamburg, Germany). Cortisol values were log transformed prior to analysis to reduce skewness. 

#### 2.3.4. Affect and Mood

Changes in affect in response to stress were assessed via the well-established Positive Affect and Negative Affect Schedule (PANAS) [40], at the same four time points as saliva collections. Changes in mood at these time points were assessed via a modified 100-point visual analog mood scale (VAMS) [41]. The VAMS consisted of three 100 mm horizontal lines, each representing a bipolar dimensional mood state: happy–sad, relaxed–tense, friendly–hostile. Participants were instructed to move the computer cursor on each line to the point that best describes their current mood state. 

### 2.4. Statistical Approach 

In order to derive a behavioral measure of CI, performance in the DD task was quantified by calculating the proportion of now choices out of the total number of choices that each participant made throughout the task (i.e., percent now choices), separately in each of the two sessions. A benefit of this measure is that it directly indexes the observed behavior, as well as that it overcomes inter-individual variability in the number of responses vs. omitted trials. The impact of stress on CI was assessed using a mixed-effect ANOVA on percent now choices, with *Group* (stress, no-stress) as a between-subject variable and *Time* (DD_1_, DD_2_) as a within-subject variable. For the group of participants that underwent stress induction, main effects of stress on physiological and subjective responses, including fluctuations in cortisol, affect and mood, were assessed using repeated measures ANOVA with the four assessment *Time* points as within-subject variables (T_−5_, T_+15_, T_+30_, T_+65_ with respect to stress onset), separately for saliva, PANAS, and VAMS, respectively. Eighteen participants were excluded from these analyses due to missing or incomplete data, leaving a final sample size of 132 participants for these analyses. Next, regression analyses were implemented in order to account for individual differences in the impact of stress on CI and assess its relation to stress-induced changes in cortisol release. For that, cortisol release throughout the four assessment time points was quantified using the area under the curve with respect to increase (AUCi) [42]. Next, in order to test a priori hypothesis regarding a potential quadratic relation between acute stress and impulsivity, and considering that AUC cannot be used to identify the exact time point throughout the study at which quadratic relations may be particularly potent, the cohort was divided into tertiles. Tertiles were divided based on the impact of stress on CI (percent now choices in DD_2_ minus percent now choices in DD_1_). ANOVAs assessing stress-induced change in saliva, PANAS, and VAMS with *Time* points as within-subject variables were repeated with the addition of *Tertile* as a between-subject variable. One-way ANOVA was used to assess the relation between *Tertiles* and cortisol AUCi. All analyses were performed with SPSS 20 (IBM) using two-tailed *p*-values. Post-hoc comparisons were performed using LSD adjustments for *p*-values. A Huynh–Feldt correction was used to account for violations of sphericity when needed. Due to the influence of the female menstrual cycle on HPA-axis function [43], data on the use of oral contraceptives (yes, no) and menstrual phase in women not taking hormonal contraceptives (follicular phase *n* = 53, luteal phase *n* = 45) were collected, and all statistical analyses were repeated while including these variables as covariates. Similarly, current smoking status (yes, no) was added as a covariate in all statistical analysis following its link to discount rates [44] and cortisol levels [45].

## 3. Results

### 3.1. Main Effects of Stress

**Figure 3:**
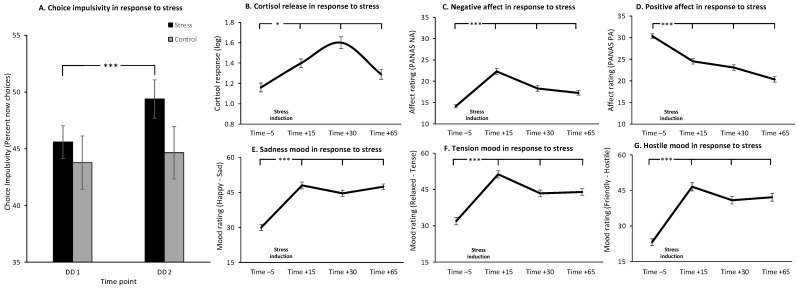
Main effects of stress. Changes in choice impulsivity (**A**), cortisol release (**B**), negative affect (**C**), positive affect (**D**), VAMS sadness scale (**E**), VAMS tension scale (**F**), VAMS hostility scale (**G**) throughout the experimental session. Across all measures, the acute stress procedure elicited the expected effects of increased impulsivity, increased cortisol release, increased negative mood and affect and decreased positive affect, at the group level. DD—delay discounting task; PANAS—positive and negative affect; VAMS—visual analogue mood scale. * *p* < 0.05, *** *p* < 0.001. Error bars represent standard error.

Assessing the impact of stress on CI (percent now choices) using mixed-effect ANOVA with *Group* (stress, no-stress) as a between-subject variable and *Time* (DD_1_, DD_2_) as a within-subject variable revealed a significant *Group* by *Time* interaction (F _(1,189)_ = 4.897, *p* = 0.030). This effect was driven by elevated CI in DD_2_ compared to DD_1_ only in the group that underwent stress induction (*p* < 0.001) (Figure 3A). For the group of participants that underwent stress induction, repeated-measures ANOVA with cortisol response to stress revealed a main effect of *Time* (F _(1.76,230.74)_ = 29.876, *p* < 0.001), due to stress-induced increase in cortisol levels from before stress (T_−5_) to 15, 30 and up to 65 min following stress offset (all *p*’s < 0.030) (Figure 3B). Repeated-measures ANOVA with affective response to stress revealed a main effect of *Time* (NA: F _(2.20,288.91)_ = 93.052, *p* < 0.001; PA: F _(2.23,292.66)_ = 209.040, *p* < 0.001), due to stress-induced increase in negative affect and decrease in positive affect from before stress (T_−5_) to 15, 30 and 65 min following stress offset (all *p*’s < 0.001) (Figure 3C,D). When considering mood ratings, repeated-measures ANOVA also resulted in a highly significant main effect of *Time* (sadness: F _(2.25,288.131)_ = 99.901, *p* < 0.001; tension: F _(2.39,306.53)_ = 61.417, *p* < 0.001; hostility: F _(2.23,285.33)_ = 108.390, *p* < 0.001). These effects were driven by an overall increase in negative mood state across all VAMS scales from before stress (T_−5_) to 15, 30 and 65 min following stress offset (all *p*’s < 0.001) (Figure 3E–G). Repeating the same analysis while controlling for oral contraceptive, menstrual phase and smoking status yielded similar results across all significant main effects and interactions (all *p*’s < 0.001).

### 3.2. Individual Differences in the Effect of Stress on CI and Stress-Induced Changes in Cortisol, Affect and Mood

**Figure 4:**
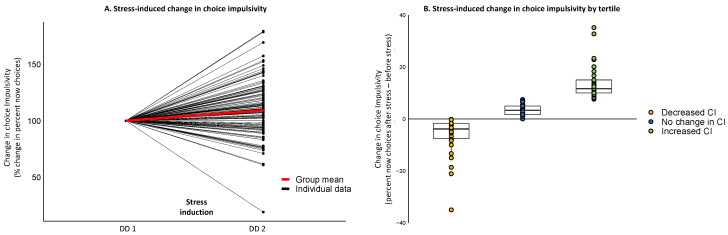
Individual differences in the effect of stress on choice impulsivity. (**A**). Individual patterns of change in choice impulsivity from before to after exposure to acute stress (*n* = 132). (**B**). Dividing the sample into tertiles based on the impact of stress on choice impulsivity yielded three sub-groups: individuals exhibiting stress-induced increase in choice impulsivity (*n* = 44), individuals exhibiting stress-induced decrease in choice impulsivity (*n* = 42), and individuals exhibiting no substantial change in their choice impulsivity following stress (*n* = 46).

Although the group mean level yielded a significant effect of stress-induced increase in CI, the magnitude and direction of the impact of stress on impulsive behavior greatly differed across participants, showing various patterns of change over time (Figure 4A). In order to account for such variability, regression analyses assessed the relation between the impact of stress on CI and stress-induced changes in endocrine responses. These analyses revealed a significant quadratic relation between cortisol AUCi and stress-induced change in CI from before to after acute stress exposure (F _(2__,129)_ = 3.463, *p* = 0.035, *r* = 0.226). In other words, high cortisol release throughout the experimental session was associated with either a decrease or an increase in CI following acute stress. In order to further pursue this, the sample was divided into tertiles based on the impact of stress on CI, yielding three sub-groups: individuals exhibiting stress-induced increase in CI (*n* = 44; mean change = 13.49 ± 5.58); individuals exhibiting stress-induced decrease in CI (*n* = 42; mean change = −6.12 ± 6.49); and individuals exhibiting no substantial change in their CI following stress (*n* = 46; mean change = 3.56 ± 2.13) (Figure 4B).

**Figure 5:**
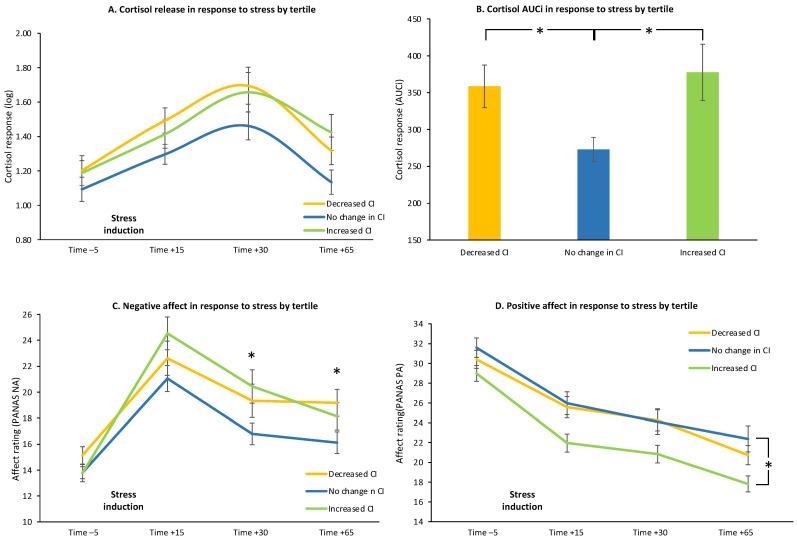
Response to stress by choice impulsivity change group. Changes in cortisol release (**A**), cortisol AUCi (**B**), negative affect (**C**) and positive affect (**D**) throughout the experimental session as a function of change in choice impulsivity group. Compared to participants that exhibited no substantial change in their choice impulsivity after stress, those that exhibited stress-induced change in their impulsive choice behavior also exhibited more stress-induced cortisol release and more negative affect, regardless of the direction of the behavioral change. *—*p* < 0.05. Error bars represent standard error.

To further investigate the obtained patterns, repeated-measures ANOVA with cortisol response to stress revealed a trend towards a significant main effect of *Tertile* group (F _(1.74,227.94)_ = 29.876, *p* = 0.097), with no *Tertile* by *Time* interaction (F _(3.55,229.31)_ = 0.803, *p* = 0.520) (Figure 5A). One-way ANOVA with cortisol AUCi values revealed a main effect of *Tertile* (F _(2__,131)_ = 3.722, *p* = 0.028), due to higher cortisol AUCi values for individuals exhibiting stress-induced increase in CI, as well as for individuals exhibiting stress-induced decrease in CI, compared to those that exhibited no substantial change in CI (*p* = 0.012, *p* = 0.041, respectively) (Figure 5B). Repeating the same analysis while controlling for oral contraceptive, menstrual phase and smoking status yielded similar results (*p* = 0.033 and *p* = 0.028, respectively). Repeated-measures ANOVA with affective responses to stress revealed a significant *Tertile* by *Time* interaction (F _(4.58,295.19)_ = 3.781, *p* = 0.004) for negative affect, due to higher negative affect for individuals exhibiting stress-induced increase in CI compared to those with no substantial change in their CI at 30 and 65 min following stress offset (*p* = 0.016 and *p* = 0.027, respectively). Higher negative affect was also present for individuals exhibiting stress-induced decrease in CI compared to those with no substantial change in CI at 65 min following stress offset (*p* = 0.012) (Figure 5C). Analysis of positive affect revealed a main effect of *Tertile* (F _(2,129)_ = 4.038, *p* = 0.021), due to decreased overall positive affect for individuals exhibiting stress-induced increase in CI, compared to those exhibiting a decrease or no substantial change in CI (*p* = 0.008 and *p* = 0.039, respectively), with no *Tertile* by *Time* interaction (F _(4.52,291.76)_ = 1.510, *p* = 0.175) (Figure 5D). Repeating the same analysis while controlling for oral contraceptive, menstrual phase and smoking status yielded similar results (*p* = 0.014 and *p* = 0.028, respectively). Analyses of mood ratings yielded no main effects of *Tertile* nor *Tertile* by *Time* interaction with and without controlling for oral contraceptive, menstrual phase and smoking status (all *p*’s > 0.070). Finally, tertile groups also did not differ with respect to trait anxiety, perceived chronic stress and trait impulsivity as assessed by the STAI-T, PSS and BIS questionnaires, respectively (all *p*’s > 0.410).

## 4. Discussion

To date, studies that assessed the impact of stress on CI have yielded mixed findings. The results of the current study may aid in resolving some of these discrepancies. Specifically, we assessed CI using the DD task in a large sample of healthy female participants that completed the task twice, before and after undergoing acute laboratory stress induction. Initial results revealed, at the group level, stress-induced increase in cortisol release and negative affect and mood, as well as in impulsive behavior, thus resembling findings from some of the previous studies in the context of stress and impulsivity. However, a more thorough examination that takes into account inter-individual variability in the impact of stress on CI revealed a quadratic relation between stress-induced change in CI and cortisol release. Similarly, dividing the sample into tertiles revealed that, compared to participants that exhibited no substantial change in their CI following stress, participants that exhibited either stress-induced increase or decrease in their CI also exhibited more stress-induced cortisol release as well as more negative affect. Taken together, these findings suggest that elevated physiological and psychological responses to stress are indeed associated with a behavioral change in the context of impulsivity, yet that such change can be expressed as either an increase or a decrease in CI.

First, it should be acknowledged that the acute laboratory stress induction procedure that was implemented here successfully induced endocrine, affective and mood responses, expressed as a highly significant increase in cortisol release, in negative affect and in negative mood, alongside a decrease in positive affect, with all of these responses lasting up to 65 min post stress induction. These findings correspond to previous studies that established the MAST as a reliable and robust laboratory procedure for acute stress induction among healthy populations [39,46,47]. Notably, the current version of the MAST includes an additional manipulation that is designed to prolong the effect of stress by denying participants’ relief from the stress until session completion [29], and indeed yielded prolonged affective and endocrine stress response patterns. Given that the temporal aspect is a critical, yet often understudied dimension of the stress response, this revised MAST manipulation could be useful in future research. This task is further useful as it enables testing of behavioral responses, in this case CI, while participants are still under rather than after stress, thus avoiding the potential biasing effect of relief that accompanies the termination of a typical acute laboratory stress induction procedure.

With specific respect to CI, current findings depict stress-induced increase in CI at the group level, expressed as more choices of an immediate smaller monetary amount compared to delayed larger amount after stress compared to before stress. This pattern of stress-induced behavioral change is consistent with some of the previous studies that implemented the DD task [16,17,18]. This effect is also in line with the more general notion that exposure to stress may lead people to act in an impulsive manner [48,49]. The fact that such a pattern was not found in the control group that completed the DD task twice without stress is aligned with the notion that DD is a stable marker of impulsivity (under no-stress conditions) [50]. Critically however, even among participants that were exposed to acute stress in the current study, individual differences emerged with respect to the magnitude and direction of the impact of stress on CI. Some participants exhibited more impulsive behavior following stress, others showed the reversed pattern, while some showed no substantial effect of stress on their impulsive behavior.

Interpreting these different patterns of behavioral responses to stress should include reference to their associated endocrine and affective response patterns. To this end, individuals that exhibited stress-induced behavioral change, regardless of the direction of the change, also exhibited more stress-induced cortisol release and more negative affect following stress. This pattern emerged as a quadratic relation between stress-induced change in CI and in cortisol release, as well as when dividing the sample into tertiles. Importantly in that regard, tertile analysis revealed that individuals in the two groups that exhibited stress-induced behavioral change demonstrated no return to pre-stress endocrine and affective levels, unlike individuals in the third no substantial change group. Such a prolonged stress response, or inability to adequately recover following stressor offset, is considered a form of stress vulnerability [51,52,53]. Accordingly, a significant change in CI following acute stress, regardless of the direction of the behavioral change, can be conceptualized as a behavioral pattern that is associated with elevated physiological and psychological responses to stress, and putatively with a tendency to exhibit stress vulnerability. With respect to elevated impulsivity, it is clear why such behavior may represent a form of stress vulnerability, and indeed numerous studies support such a scenario [54,55,56]. Reduced impulsivity after stress, nevertheless, is not often reported, nor is it typically considered a vulnerability marker. Following current results, we can speculate that reduced impulsivity following stress may also represent a form of stress vulnerability. To this end, an adaptive response to stress exposure should involve some recognition that current existing resources will not last for long, and that the future is more uncertain than one has thought before. This in turn may provide some rationale as to why increasing the likelihood of selecting future rewards instead of immediate rewards post-stress could be considered maladaptive. These speculative notions are further supported, in a broader context, by the repeated demonstrations of amplified but also blunted physiological and endocrine stress response patterns among stress-related psychopathological populations [26,27,28]. Among healthy adults, quadratic relations were demonstrated between cortisol and subjective responses to acute stress, such that both hyper and hypo cortisol responses were associated with elevated negative affect compared to moderate cortisol response [29]. In another study, slightly elevated levels of cortisol after acute stress induction were associated with improved cognitive performance, while highly elevated levels of cortisol were associated with impaired cognitive performance [32]. Taken together, current results contribute to the notion that exaggerated behavioral, physiological, endocrine and affective response to stress might be associated with a negative outcome, yet that the same could also be relevant in the case of blunted responses across domains. This notion is further in line with the inverted U shape concept of stress that claims that a mild-to-moderate stress response may carry beneficial effects, yet an extreme response, either complete absence of stress response, or an excessive and prolonged response may carry deleterious effects [51]. Interestingly, in the two studies mentioned above, as in the current study, quadratic patterns emerged specifically among female participants, thus raising the speculative idea that the potency of the inverted U pattern in the context of stress is gender-dependent.

It should be noted, however, that the current study included only healthy young females. The inclusion of females only in this study can be seen as both a strength and a limitation. Gender differences have been observed in studies that assessed the effect of acute stress on decision-making including in the context of CI [20,30,31,32,33]. Accordingly, including only females reduces substantial gender-dependent variability, yet at the same time, limits the generalizability of current results. Future studies could assess whether similar patterns of stress-induced change in CI also emerge among healthy males, as well as among other age groups and psychopathological populations. Interestingly, within our cohort, the trait measures of anxiety, impulsivity and perceived chronic stress were not associated with individual differences in the impact of acute stress on impulsive behavior. Other social, environmental and personal factors that were not assessed in here may have contributed to variability in the impact of acute stress on CI. One prominent factor is socioeconomic status, a key element of allostatic load [57], that was shown before to directly impact discounting rate [58]. Furthermore, given that in the current study the impact of stress on CI was assessed shortly after stress, future studies should also assess whether the observed behavioral effects are long lasting, for instance whether differences in the impact of acute stress on CI can also be detected days or even months after stress offset. One additional limitation of the current study is the decision to use strict tertile criteria and define increased and decreased stress-induced CI as the upper and lower 33% in terms of change in DD performance. Given a larger and more heterogonous cohort, the application of data-driven approaches could have optimized group classification. Finally, performance in the DD task was quantified using percent now choices, a relatively simple measure compared to additional existing measures for CI, such as area under the curve (AUC) derived from indifference points based on logistic regression, and discount factor (k) as the hyperbolic function slope [59]. Critically, reliable calculation of these additional measures requires a significant number of trials and ideally even repetition of identical delay by number of combinations, as those models are sensitive to wrong choices. Our task included only 60 non-repeating trials, and for a large proportion of participants, these measures yielded invalid results or no measures at all, as models did not converge. Therefore, percent now was used as a single quantified behavioral measure that is adjusted to the layout of the task.

## 5. Conclusions

Taken together, our results reveal robust behavioral, endocrine and affective response to acute stress at the group mean level, expressed as an increase in CI, cortisol release and negative affect, respectively. By that, current results are in line with a substantial crop of evidence that suggests that stress may impact decision-making [3,32,60], and in particular may yield an increased tendency to act in an impulsive manner [17,18]. Critically, however, a more thorough examination that takes into account inter-individual variability in the impact of stress on CI revealed that, compared to participants that exhibited no substantial change in their CI after stress, participants that exhibited either stress-induced increase or decrease in their CI also exhibited more stress-induced cortisol release and more negative affect. These findings may explain some of the discrepancies in the literature on the interaction of stress and impulsivity, as most reports focused on group mean effect or assessed linear or binary relations. Instead, current findings suggest that elevated physiological and psychological responses to stress can be associated with either an increase or a decrease in CI, thus depicting quadratic relations between stress and impulsivity, in accordance with the inverted U shape concept of stress. Considering that impulsivity and stress are known vulnerability factors for addiction and relapse [61,62], insight derived from the present study may provide vital information towards improved understanding of the role of stress in sub-optimal choice selection among healthy females in the context of prevention of substance abuse and other disorders of impulsivity.

## Data Availability

The data collected in this study are available from the corresponding author upon request.
